# Over-Expression of CD200 Protects Mice from Dextran Sodium Sulfate Induced Colitis

**DOI:** 10.1371/journal.pone.0146681

**Published:** 2016-02-03

**Authors:** Zhiqi Chen, Kai Yu, Fang Zhu, Reginald Gorczynski

**Affiliations:** Transplant Research Division, The Toronto Hospital, Department of Surgery and Immunology, University Health Network, University of Toronto, Toronto, Canada; Massachusetts General Hospital, UNITED STATES

## Abstract

**Background and aim:**

CD200:CD200 receptor (CD200R) interactions lead to potent immunosuppression and inhibition of autoimmune inflammation. We investigated the effect of "knockout"of CD200 or CD200R, or over-expression of CD200, on susceptibility to dextran sodium sulfate (DSS)—induced colitis, a mouse model of inflammatory bowel disease (IBD).

**Methods:**

Acute or chronic colitis was induced by administration of dextran sodium sulfate (DSS) in four groups of age-matched C57BL/6 female mice: (1) CD200-transgenic mice (CD200^tg^); (2) wild-type (WT) mice; (3) CD200 receptor 1-deficient (CD200R1KO) mice; and (4) CD200-deficient (CD200KO) mice. The extent of colitis was determined using a histological scoring system. Colon tissues were collected for quantitative RT-PCR and Immunohistochemical staining. Supernatants from colonic explant cultures and mononuclear cells isolated from colonic tissue were used for ELISA.

**Results:**

CD200KO and CD200R1KO mice showed greater sensitivity to acute colitis than WT mice, with accelerated loss of body weight, significantly higher histological scores, more severe infiltration of macrophages, neutrophils and CD3^+^ cells, and greater expression of macrophage-derived inflammatory cytokines, whose production was inhibited in vitro (in WT/CD200KO mouse cells) by CD200. In contrast, CD200^tg^ mice showed less sensitivity to DSS compared with WT mice, with attenuation of all of the features seen in other groups. In a chronic colitis model, greater infiltration of Foxp3^+^ regulatory T (Treg) cells was seen in the colon of CD200^tg^ mice compared to WT mice, and anti-CD25 mAb given to these mice attenuated protection.

**Conclusions:**

The CD200:CD200R axis plays an immunoregulatory role in control of DSS induced colitis in mice.

## Introduction

Inflammatory bowel disease (IBD) is an autoimmune-like inflammatory disorder of the gastrointestinal tract comprising two major forms, Crohn’s disease (CD) and ulcerative colitis (UC) [1]. Although the etiology of IBD remains unclear, a combination of genetic susceptibility along with intestinal microbial and environmental factors is thought to contribute to an imbalance of the mucosal immune system, resulting in the overproduction of inflammatory cytokines and infiltration of myeloid cells and lymphocytes, with subsequent initiation of disease [2,3].

Genetic susceptibility in the pathogenesis of IBD has been documented-see review [4]. Multiple cytokines produced by different subsets of T-helper cells are also implicated in the pathogenesis of IBD including, interferon-γ (IFN-γ), interleukin (IL)-4 and IL-17/IL-22, produced by Th1, Th2 and Th17 cells respectively. Other cytokines thought to play a role in the development of IBD, are TNF-α, IL-6 and IL-12 [5]. A potential contributory role for a member of a family of hematopoietic effector cells called innate lymphoid cells (ILCs) in IBD has also been suggested- group 3 ILCs, a retinoic acid receptor-related orphan receptor γt (RORγt)-dependent population which produces Th17 like cytokines, is now implicated in development of intestinal immune response [6,7]. Type 1 membrane proteins implicated in regulation of autoimmunity, including the T cell immunoglobulin and mucin domain (Tim-3), have also been reported to regulate the development of IBD [8].

CD200 is a type I membrane protein belonging to the immunoglobulin supergene family. This molecule plays an immunoregulatory role following interaction with its receptors, CD200Rs [9]. Previous studies have shown that CD200 enhanced graft survival [10], reduced spontaneous fetal loss [11] and increased growth of tumor cells [12,13]. CD200 overexpression exerts a protective function in autoimmune inflammation in models of autoimmune encephalomyelitis (EAE) [14], autoimmune uveoretinitis (EAU) [15], collagen-induced arthritis [16] and inflammation-mediated neurodegeneration [17]. The role of CD200 in the pathogenesis of IBD, another autoimmune-like inflammatory disease with high morbidity, has not been fully evaluated.

Dextran sodium sulfate (DSS) is a commonly used chemical for induction of murine colitis [18] that has many similarities to human IBD. In the studies described below we examined the role of CD200 in DSS-induced colitis using doxycycline (Dox)-inducible CD200^tg^ mice, as well as CD200R1KO and CD200KO mice. We show that by comparison with wild-type (WT) control mice, increased expression of CD200 provides protection from both acute and chronic colitis after DSS-induction, whereas severe colitis was seen in mice lacking either CD200 or CD200R1.

## Materials and Methods

### Mice

C57BL/6 female mice, with high susceptibility to DSS-induced colitis, were used throughout. Wild-type mice were purchased from the Jackson laboratories (Bar Harbor, ME). Homozygous rtTA2^s^-M2 CD200^tg^ mice, CD200KO and CD200R1KO mice, all on a C57BL/6 background, were generated in our laboratory as described previously [19–21]. All mice were housed five per cage under specific pathogen-free conditions, allowed standard diet and water ad libitum, and used at 6–8 wk of age. All mice received Dox in their drinking water (1 μg/ml) for 7 days before DSS induction-this induces CD200 over-expression in CD200^tg^ mice [20].

Animal experimentation was performed following guidelines of an accredited animal care committee (protocol no. AUP.1.5). Humane endpoints were used in all studies, with mice monitored daily. Animals were euthanized (overdose with pentobarbital) when they were exhibiting signs of distress (weight loss≥25%; hunched posture; diaorrhea; loss of active movements). As noted below, mortality was seen only in studies of acute colitis, with mean (averaged over ~120 mice) ≤15%. Animals with diaorrhea (but weight loss <25%) received daily ip injections of saline (1ml x3 at 8hr intervals) to avoid dehydration.

### Induction of colitis

Acute DSS colitis was induced by giving mice distilled drinking water containing 3% (wt/vol) DSS (m.w. = 40 kDa; ICN Biochemicals, Aurora, OH) for 7 days [22]. For induction of chronic colitis, mice were treated with 5 days of 3% DSS followed by 7 days of normal drinking water for a total of 3 cycles. Dox was injected into all mice daily during DSS administration by i.p. injection at a concentration of 0.5 mg/kg mouse body weight. Body weight was measured three times/week throughout the experiment. The maximum mortality observed following induction of acute colitis (seen in CD200KO/CD200RKO mice) was 15% averaged over all studies (>40 mice/group), while no significant mortality was seen any groups in the chronic colitis model. As described in subsequent Figs (later) maximum weight loss in both models was ~25% (again in CD200KO and CD200RKO mice), with all mice suffering from acute colitis recovering weight loss within 7–10 days post cessation of DSS exposure. In the chronic colitis model, CD200KO and CD200RKO mice, while recovering weight post DSS exposure, were still some 10–15% below starting weight 5 weeks following initial treatment.

Where CD4^+^T cells were depleted from mice with acute DSS-induced colitis, mice received 50mg/mouse of anti-mouse CD4 mAb (GK1.5), purchased from Cedarlane Labs, Hornby, Ontario, iv at 4 and 7 days post DSS initiation, and were sacrificed at 9 days post DSS initiation. In a chronic colitis model anti-CD4 mAb was given three times (1 day following completion of each DSS treatment). PC61, an anti-CD25 mAb (to deplete Foxp3^+^ Tregs) was given to some groups on the same schedule. All animals were sacrificed by cervical dislocation under halothane anaesthesia.

### Histological analysis of colitis

Colonic fragments from the proximal and distal parts (0.5 cm) of the colon were fixed in 10% neutral-buffered formalin for 24 h. Paraffin cross-sections (5 μm) were prepared and stained with hematoxylin and eosin. Colon sections were scored using the scoring system for inflammation-associated histological changes in the colon [18]. For the acute colitis model, tissue was harvested 9 days post initiation of DSS treatment. For the chronic colitis model tissue was harvested 8 days after initiation of the third DSS treatment-these same tissue explants were used for immunohistochemistry (below).

### Immunohistochemistry

Colonic fragments from all experimental group were fixed in 10% neutral-buffered formalin for 24 h and stained with rat anti-mouse F4/80 (Serotec, Raleigh, NC), Gr1 (Cedarlane, Burlington, ON), CD3 or FoxP3 (San Diego, CA) monoclonal antibody, respectively, followed by incubation with biotin conjugated rabbit anti-rat IgG (Vector Laboratories, Burlington, ON). DAB (3,3’-diaminobenzidine) was used for colour development. The slides were counterstained with Harris’ Hematoxylin. For comparison of cell infiltration in tissues from different groups of mice a total of ~1000 cells (x400) were enumerated, using 4–5 slides/group, to assess % F4/80;Gr1, CD3 or Foxp3 staining [23,24].

### RNA isolation and real- time RT-PCR

Total RNA was isolated from colonic tissue using TRIzol reagent (Invitrogen Canada, Burlington, ON). Total RNA (1 μg) was treated with DNase I and then reverse transcribed using High Capacity cDNA Reverse Transcription Kit (Applied Biosystems, Foster city, CA) following the manufacturer’s instruction. First strain cDNA was diluted 1:20 and used for quantitative PCR on an ABI 7900HT Sequence Detection system with SYBR green master mix (Applied Biosystems). Messenger RNA levels were normalized to a composite of GAPDH and HPRT expression levels. Experiments were repeated three times with three independent cDNA syntheses. The primers used for real-time RT-PCR are shown in Table 1.

**Table 1:** Primers used for real- time PCR.

IFN-γ	forward: 5'-TATTGCCAAGTTTGAGGTCAACA-3ʹ
	reverse: 5’-GCTGGATTCCGGCAACAG-3’
TNF-α	forward: 5'-AGACCCTCACACTCAGATCATCTTC-3’
	reverse: 5’-CCACTTGGTGGTTTGCTACGA- 3’
IL-1β	forward: 5’-TCGTGCTGTCGGACCCATAT-3’
	reverse: 5’-GGTTCTCCTTGTACAAAGCTCATG-3’
IL-4	forward: 5’-TCATCGGCATTTTGAACGAG-3’
	reverse: 5’-TTTGGCACATCCATCTCCG-3’
IL-6	forward: 5’-CTCTGGGAAATCGTGGAAATG-3’
	reverse: 5’-CAGATTGTTTTCTGCAAGTGCAT-3’
IL-10	forward: 5’-AAGGCAGTGGAGCAGGTGAA-3’
	reverse: 5’-TTCTATGCAGTTGATGAAGATGTCAA-3’
IL-12	forward: 5’-CCCAAGGTCAGCGTTCCA-3’
	reverse: 5’-GGCAAGGGTGGCCAAAA-3’
IL-17	forward: 5’-CTCAGACTACCTCAACCGTTCCA-3’
	reverse: 5’-CCAGATCACAGAGGGATATCTATCAG-3’
IL-22	forward: 5’-GTGCCTTTCCTGACCAAA-3’
	reverse: 5’-TCTCCTTCAGCCTTCTGA-3’
IL-23	forward: 5’-GACAACAGCCAGTTCTGCTT-3’
	reverse: 5’-AGGGAGGTGTGAAGTTGCTC-3’
IL-23R	forward: 5’-AATTTGACGCCAATTTCACA-3’
	reverse: 5’-ACCAGTTTCTTGACATCGCA-3’
TGF-β	forward: 5’-CGAAGCGGACTACTATGCTAAAGA-3’
	reverse: 5’-GTTTTCTCATAGATGGCGTTGTTG-3’
Foxp3	forward: 5’-AGTCTGCAAGTGGCCTGGTT-3’
	reverse: 5’-GGGCCTTGCCTTTCTCATC-3’
CCR4	forward: 5’-AGACTGTCCTCAGGATCACTTTCA-3’
	reverse: 5’-CCGGGTACCAGCAGGAGAA-3’
CCL-17	forward: 5’-ATGCCATCGTGTTTCTGACTGT-3’
	reverse: 5’-GCCTTGGGTTTTTCACCAATC-3’
CCL-22	forward: 5’-AAGCCTGGCGTTGTTTTGAT-3’
	reverse: 5’-TCCCTAGGACAGTTTATGGAGTAGCT-3’
T-bet	forward: 5’-CCAGGGAACCGCTTATATGT-3’
	reverse: 5’-CTGGGTCACATTGTTGGAAG-3’
RORα	forward: 5’-ACCCGAACCCATATGTGACT-3’
	reverse: 5’-TTTGGATATGTTCTGGGCAA-3’
RORγt	forward: 5’-CAGAGGAAGTCAATGTGGGA-3’
	reverse: 5’-ATGATCTGGTCATTCTGGCA-3’
GATA3	forward: 5’-ACGCTCCTTGCTACTCAGGT-3’
	reverse: 5’-ACTGCACACTGATTCCTTGG-3’
NKp46	forward: 5’-TCTGGTCAAAGTCGAGCAAC-3’
	reverse: 5’-CCAAGGTTACCTCAGGCTG-3’
AHR	forward: 5’-ACATCGACATAACGGACGAA-3’
	reverse: 5’-AAGCCGAGTTCAGCAAAGTT-3’

### Colonic explant culture and multi-analyte Elisarray

Colons were removed, washed three times with cold PBS containing 100 IU penicillin and 100 μg/ml streptomycin, and opened longitudinally. The proximal and distal parts of the colon were cut into strips 3 mm in length and cultured in 500 μl of supplemented RPMI 1640 culture medium at 37°C with 5% CO_2_ humidified air for 24 h following which supernatants were collected and particulate material removed by centrifugation for 10 min at 1000 x g. The supernatants were subsequently used for cytokine and chemokine analysis using a Multi-analyte Elisarray kit (Qiagen, Mississauga, ON). Twelve proinflammatory cytokines and chemokines were examined in the supernatants, including IL-1β, IL-4, IL-6, IL-10, IL-12, IL17A, IFN-γ, TNF-α, TGFβ, MCP1, MIP-1α and MIP-1β. Capture antibodies for the 12 cytokines and chemokines were coated on one Elisarray microplate and 50 μl of samples were added to the wells of the plate. After 2 h incubation and exhaustive washing to remove unbound proteins, 100 μl of biotinylated detection antibodies were added. Thereafter, an avidin-horseradish peroxidase conjugate was added after 1 h incubation. After further washing substrate solution was added. A stop solution was added after 30 min and the absorbance at 450 nm was read. The manufacturer’s computer software, which includes relevant cytokine controls, was used to express the cytokine “signals” in different samples relative to one control group (nominally scored as = 1-see Fig legends 3C and 6C).

### Preparation of Lamina Propria (LP) cells from colonic tissue

Small strips (~0.4cm) of colon isolated as above from pools of 2–3 mice/group were incubated in 15ml of predigestion PBS solution with 5mM EDTA and 20mM HEPES(PH = 7.2) for 15 minutes at room temperature with constant stirring using a magnetic bar **(**~50 rpm/min). Strips were harvested, resuspended in more of the same fresh medium, and the process repeated x3. After the final wash, samples were in 20ml of digestion medium (alpha MEM with 20%FCS containing 2mg/ml collagenase Type3 and 0.2mg/ml DNase I (both from Sigma biochemical, Mississauga, Ontario, Canada)) at 37 ^o^C for 60 min, again stirring at 50rpm. The supernatant containing LP cells was retained in a 50ml tube, and the digestion process repeated x2 with fresh digestion medium on each occasion. The combined supernatants were strained through a 70um cell strainer, centrifuged for 5 mins at 1300rpm at 4°C, and resuspended in 5ml PBS containing 0.5%BSA and 2mMEDTA for counting and subsequent experimentation as described.

In some studies described in the text, CD11c^+^, CD11b^+^ and CD4^+^ cell populations were isolated directly for analysis of cytokine production (using commercial ELISA assays: BioLegend: USA) using CD11c, CD11b or CD4 magnetic-activated cell sorting MACS beads (Miltenyi Biotech, USA) [25]. Where CD200Fc was added to these cultures the final concentration used was 5mg/ml.

### FACS analysis

CD4-PE-Cy7, CD3-FITC, Foxp3-PE, Gr1-FITC and F4/80-FITC (Biolegend, San Diego, USA) were used to identify different cell populations on a BD LSR flow cytometer. Cell surface staining and intracellular staining were conducted according to the mAb manufacturer’s instructions, using LP cells isolated as above.

### Statistical analysis

Statistical significance was determined using the Student *t* test or one-way ANOVA followed by Tukey tests. P-values less than 0.05 were considered statistically significant and shown in Figs. In studies comparing weight loss curves in different groups of DSS-treated mice receiving anti-CD4/anti-CD25 mAbs, curves were compared using Mann-Whitney U-tests.

## Results

### Overexpression of CD200 attenuates body weight loss in DSS-induced acute colitis

**Fig 1:**
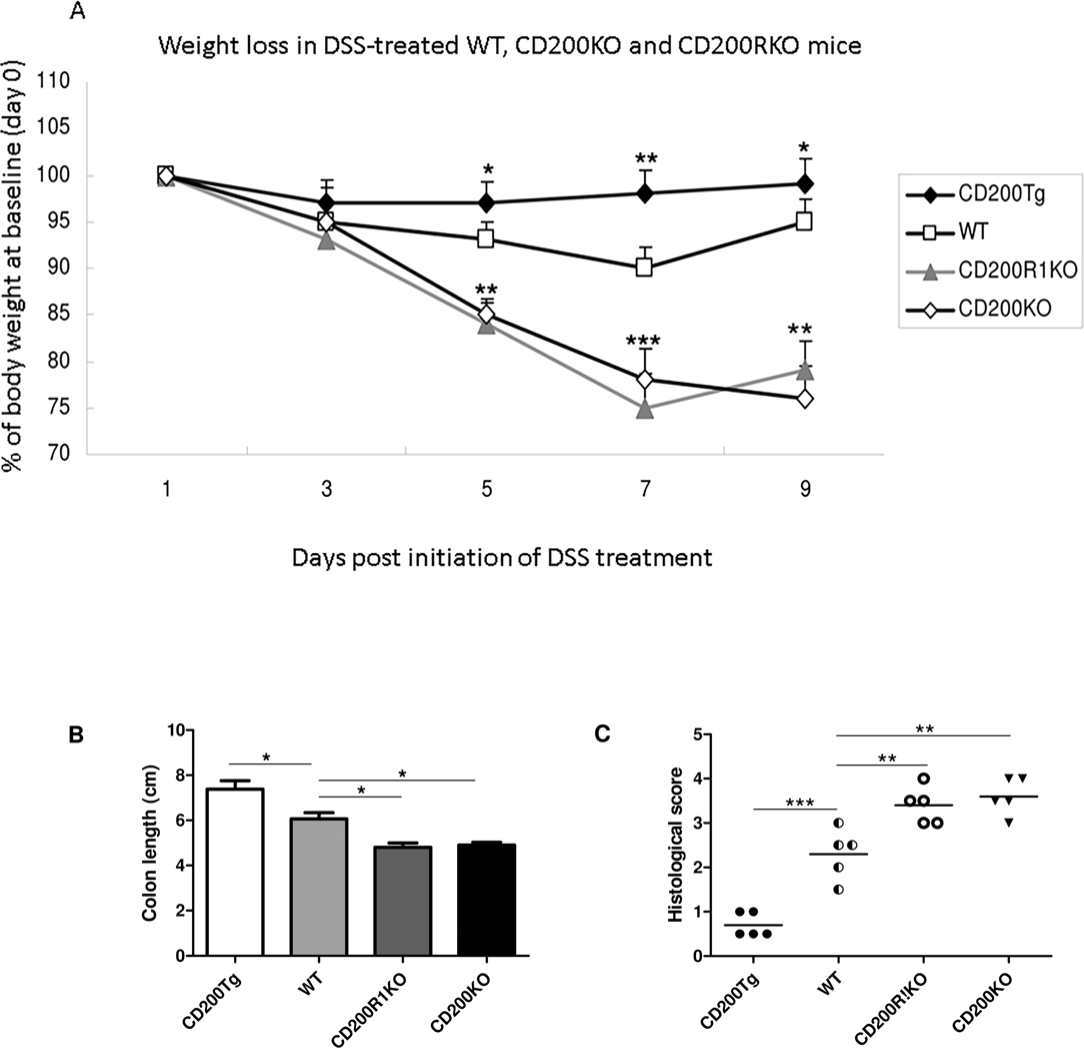
Changes in body weight, colon length and histological score of CD200^tg^, WT, CD200R1KO and CD200KO mice with DSS-induced acute colitis. (A) Change in body weight. Percent weight loss from baseline (y-axis) is plotted versus time post initiation of DSS treatment (x-axis). Data points represent mean ± SD for 5 mice. (B) Change in colon length assessed on day 9 following initiation of DSS treatment for the same same mice as in panel A. Data are expressed as mean ± SD of five mice. (C) Change in histological score (again day 9 post start of DSS) for mice of panel A. All independent experiments were performed three times, yielding similar results. * p < 0.05; ** p < 0.01; *** p < 0.001 compared with WT controls on the same day.

To explore a role for CD200 in the pathogenesis of acute colitis, we exposed C57BL/6 WT, CD200^tg^ mice, CD200R1KO mice and CD200KO mice to 3% DSS in their drinking water for 7 days followed by oral administration of DSS free water for 2 days. Body weights for the four groups were recorded for 9 days (Fig 1A), with significant differences detected by day 5–7. WT mice had lost ~ 10% of the body weight by day 7, with CD200R1KO and CD200KO mice losing 20 ~ 25% of body weight over the same time, while CD200^tg^ mice were fully protected from weight loss (Fig 1A). Colon length, as a surrogate marker for colon injury, was also measured in the four groups. Again CD200^tg^ mice treated with DSS showed significant attenuation of change relative to untreated control animals, with markedly reduced colon length seen in WT, CD200R1KO and CD200KO mice (Fig 1B). These results support the hypothesis that CD200 expression can regulate the severity of DSS-induced acute colitis. In additional studies (data not shown) we observed minimal reproducible changes in mRNA expression for CD200 or CD200R in isolated colonic tissue from WT animals from day 0 to day 7 post DSS (≤ 1.5-fold increase in CD200 expression at 6 days post initiation of DSS). However, and as published elsewhere [19,20], CD200^tg^ mice showed ubiquitous marked over-expression of CD200mRNA (≥10-fold) in all cells, at all time points, with no detectable CD200 expression in CD200KO mice. CD200KO showed inconsistent moderately elevated (≤1.5 fold) CD200R mRNA expression relative to WT controls, while CD200RKO mice showed absent CD200R mRNA expression, with equivalent CD200 expression to that of WT controls [21].

### CD200 reduced the severity of histological changes in DSS-induced acute colitis

The histological scores for the four groups in Fig 1A and 1B are shown in Fig 1C (tissues assessed at 9d post DSS). Among the four groups, the score for the CD200R1KO or CD200KO group was significantly higher than that of either WT group (p<0.01) or CD200^tg^ group (p<0.01). Comparing WT and CD200^tg^ groups, the score of the CD200^tg^ group was significantly lower than that of WT group (p<0.01).

**Fig 2:**
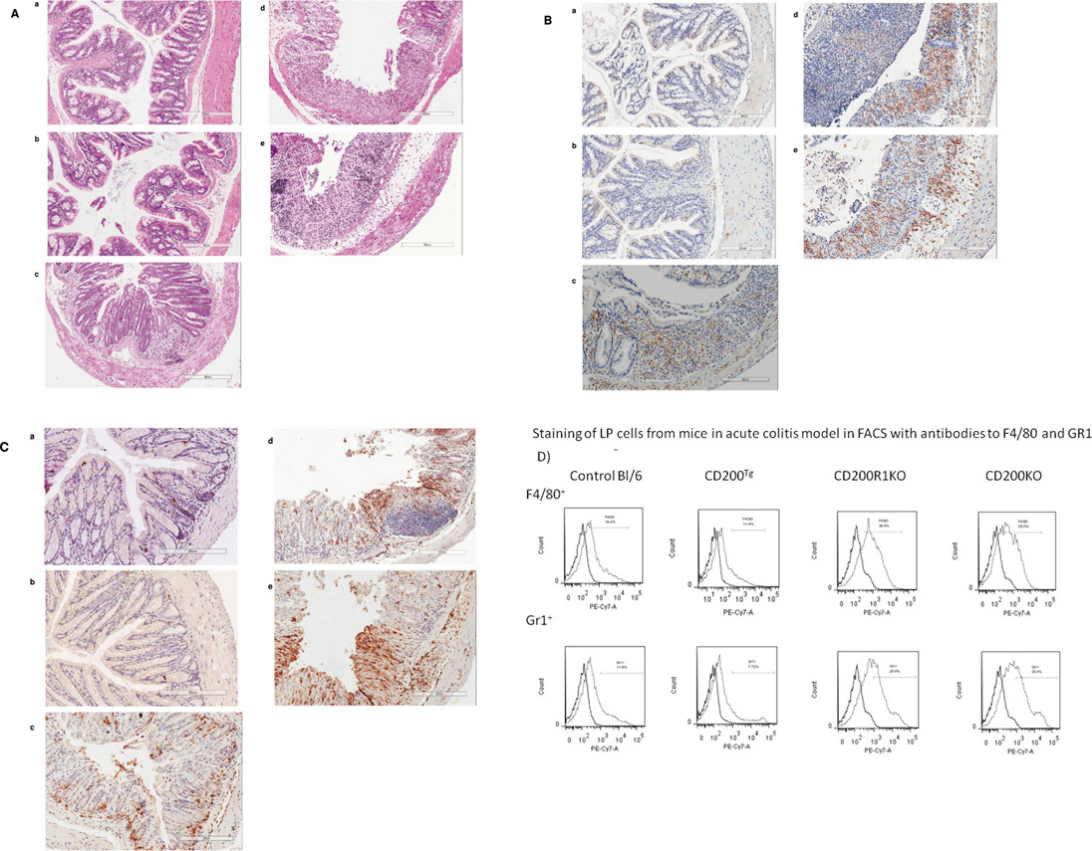
Effect of CD200 on histological changes and cell infiltration in colonic tissue from mice with DSS-induced acute colitis. Sections from colon (day 9 post start of DSS) of control (*a*), CD200^tg^ (*b*), WT (*c*), CD200R1KO (*d*) and CD200KO (*e*) mice were stained with (A) H&E, or with (B) rat anti-mouse F4/80, or (C) rat anti-mouse Gr1 mAbs, respectively. Percent staining in tissue is shown in Table 1, counting 1000 cells for each group using 5 slides/group. (D) FACS staining with anti-F4/90 or anti-GR1 mAb using isolated LP cells obtained from the groups of mice documented above. Control cells were stained with Fluorochrome labeled mouse Ig.

Loss of crypts, infiltration of inflammatory cells in the mucosa and submucosa, edema of the submucosa, ulceration and erosion are common features in colitis. As shown in Fig 2A, crypt loss and infiltrating leukocytes were seen in the colons of WT, CD200R1KO and CD200KO groups (day 9 post DSS). The most marked histological changes were observed in CD200R1KO and CD200KO mice with transmural inflammation and loss of both the crypts and the epithelial cell layer. Few histological changes compared to control mice were detected in the colons of the CD200^tg^ group.

The major cells implicated in acute inflammation in IBD include macrophages and neutrophils [26]. In CD200R1KO and CD200KO mice, tissue infiltrating F4/80^+^ (Fig 2B) and Gr1^+^ (Fig 2C) cells were increased relative to WT, while in all three groups substantial increases were seen relative to CD200^tg^-see also Table 2. Note that there is evidence that F4/80 stains both tissue eosinophils and macrophages, while Gr1 detects both neutrophils and monocytes (Ly6C and Ly6G) [23,24]. Nevertheless these data are consistent with the hypothesis that acute DSS-induced colitis is mainly driven by innate immune mechanisms [27], and that CD200 can suppress the acute inflammatory response in association with reduction in infiltration of inflammatory cells. These data were confirmed by FACS staining of LP cells from the same animals (Fig 2D).

**Table 2:** Quantitation of staining of F4/80^+^ and GR1^+^ cells in slides shown in Fig 2B and 2C.

Cell staining	Control	CD200^tg^	WT	CD200R1KO	CD200KO
%anti-F4/80	0.5±1	4+2	18±6*	35±7**	32±7**
%anti-Gr1	1.5±1	3+2	13±5*	27±7**	30±6**

*P<0.05 compared with CD200^tg^

** p<0.05 compared with WT

***Footnote*:** Colonic sections from 5 different groups of mice were stained with anti-F4/80 or anti-GR1 antibodies. Percent cells stained was enumerated by counting (5 slides/group) a total of 1000cells.

### Effect of CD200 on cytokine production in DSS-induced acute colitis

The role of cytokines in the immunopathological manifestations of inflammatory bowel disease has been documented, with multiple proinflammatory cytokines shown to be elevated in colonic tissue [5]. To investigate whether CD200 affects the production of cytokines and chemokines in the experimental mice described we performed real time RT-PCR and multi-analyte Elisarray to compare the expression level of cytokines and/or chemokines in the four experimental groups of colitis.

**Fig 3:**
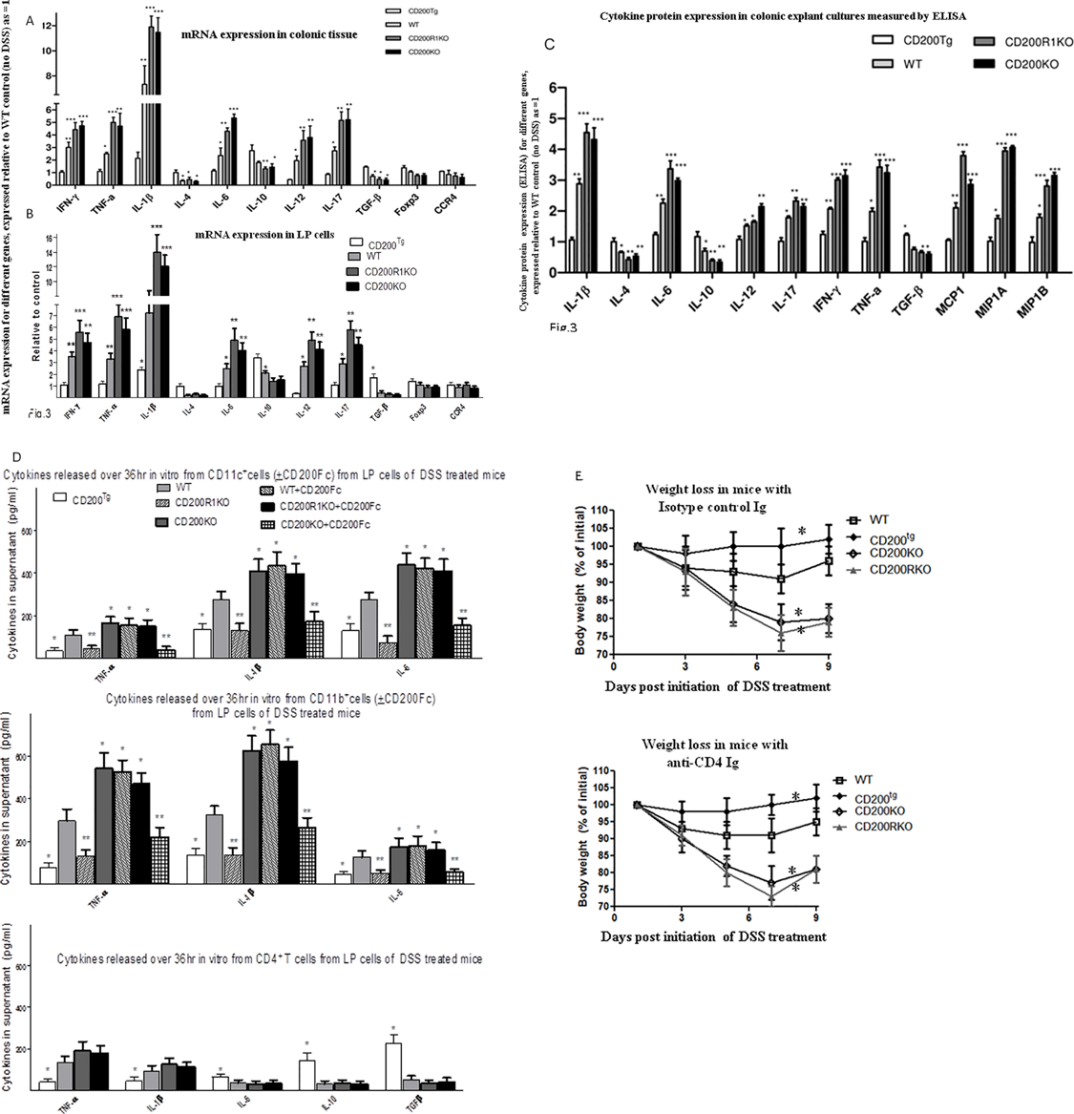
Effect of CD200 on expression of multiple cytokines and in DSS-induced acute colitis. Cytokines and chemokines were analyzed in colonic tissue from CD200^tg^, WT, CD200R1KO and CD200KO mice with DSS-induced acute colitis. A and B, Real-time RT-PCR was performed using total RNA extracted from colonic tissue (5 samples/group) or LP cells (3 samples/group), with data normalized to the expression of GAPDH and HPRT in the same organ. Expression levels in CD200^tg^, CD200R1KO and CD200KO mice (mean ± SD in 5 mice) are shown relative to that in WT at day 0 (designated as 1). * p < 0.05; ** p < 0.01; *** p < 0.001 compared to WT control (day 0). (C) Tissue explants from the four experimental groups were cultured and used for multi-analyte Elisarray. Again, relative expression levels of cytokine/chemokine (mean ± SD in 5 mice) in CD200^tg^, CD200R1KO and CD200KO mice are shown compared to that in WT at day 0 (designated as 1), using the manufacturer’s software supplied. * p < 0.05; ** p < 0.01; *** p < 0.001, compared to this WT control (day 0). (D) Inflammatory cytokines measured by commercial ELISA (Biolegend: USA) in 36hr culture supernatants of colon-derived CD11c^+^, CD11b^+^, or CD4^+^ T cells (isolated by MACS columns) from WT, CD200^tg^, CD200KO or CD200RKO mice 9 days post initiation of DSS treatment. Cells were obtained from a pool of 4 mice/group, and cultured without further stimulation for 36hrs in complete medium. In some cases, as shown, cells were cultured in the presence of CD200Fc (5mg/ml). Data show mean (+SD) of triplicate measurements. *, indicates p<0.05 compared with WT control; **indicates p<0.05 compared to equivalent group with no CD200Fc. E, Comparison of weight loss in 4 groups of DSS treated mice receiving anti-CD4mAb (right hand panel) or isotype control Ig (left hand panel)-see also Fig 1. Data are for 4 mice/group, given 50mg Ig at days 4/7 post initiation of DSS treatment. Comparison of equivalent groups in right/left hand panels (Mann Whitney U-test) revealed no statistically significant differences.

As shown in Fig 3A, using real time RT-PCR with RNA extracted from colonic tissue, the expression of mRNAs for genes encoding Th1/Th17 cytokines and other proinflammatory cytokines, including IFN-γ, IL-1β, IL-6, IL-12, IL-17 and TNF-α, were significantly increased in tissue from both CD200R1 and CD200KO mice compared with WT. CD200^tg^ mice displayed higher levels of mRNAs for the Th2 cytokines IL-4, IL-10 and the anti-inflammatory cytokine TGF-β, with significantly lower levels of mRNAs for macrophage-derived and Th1/ Th17 cytokines compared with other groups-similar patterns were seen using the same cytokine primers with mRNA from isolated LP cells (Fig 3B).

Essentially equivalent cytokine patterns were also observed when cytokine protein expression levels were compared in isolated colonic tissue (Fig 3C) using multi-analyte ELISA kits-the manufacturer’s software was used to compare different cytokines referenced to one source as control. Our earlier study had shown that increased expression of CD200 was associated with polarization of cytokine production to type-2 cytokines [28]. Similar polarization of cytokine production following CD200 overexpression was also seen in this IBD model, with evidence for Th1/Th17 responses in WT, CD200R1 and CD200KO groups. Compared with WT mice, both CD200R1KO and CD200KO mice displayed higher production of several major inflammatory mediators, including IL-1β, IL-6, TNF-α, monocyte chemotactic protein 1 (MCP1), macrophage inflammatory protein (MIP) -1 α and β (Fig 3C), supporting conclusions resulted from histology samples that myeloid cells played an important role in the pathogenesis of acute inflammatory response.

Dendritic cells (DCs) have been implicated in adoptive transfer of DSS colitis in mice (with attenuation of disease in mice in which DCs were depleted) [29]. We next investigated whether CD11c^+^, CD11b^+^ or CD4^+^ T cells, enriched on MACS-columns were the prominent source of the inflammatory cytokines detected in colonic explants-note that anti-CD11c/-CD11b do not absolutely discriminate intestinal DCs/macrophages [24]. In some cultures, exogenous CD200Fc (5mg/ml) was included in vitro to suppress cytokine production [21, 28]. Data shown in Fig 3D, using individual quantitative commercial ELISA kits to measure cytokines from LP cells (one of two studies) provide additional information to those obtained from explant cultures. In particular, while both CD11c^+^ and CD11b^+^ cells from DSS treated CD200^tg^ mice release minimal amounts of cytokines into culture over 36hrs, the greatest levels of IL-1b and TNFa were released from CD11b^+^ cells of CD200KO and CD200R1KO mice, while CD11c^+^ cells of these same mice were the best source of IL-6, and to a lesser degree, IL-1b also. CD4^+^ cells from all groups were not a major source of the inflammatory cytokines produced, but those from CD200^tg^ mice did contribute significantly to IL-10 and TGFb production. Furthermore, exogenous addition of CD200Fc to cultures of both CD11b^+^ and CD11c^+^ cells of WT and CD200KO mice attenuated cytokine production (to levels approaching those observed for CD200^tg^ mice), with, as anticipated, no effect on levels released by CD200RKO mice.

ILCs are also implicated in intestinal homeostasis[6,7,30]. Preliminary mRNA expression analysis on tissue isolates (S1A and S1B Fig and S2A and S2B Fig) for mice with acute (and chronic-see later) colitis respectively showed altered expression of mRNA for many genes characteristic of ILCs in CD200R1KO and CD200KO compared to WT mice.

While data in Fig 3D suggest CD4^+^ T cells were not a major source of inflammatory cytokines associated with acute colitis, we performed additional studies to explore the importance of T cells in vivo in this model. FACS staining (not shown) showed no difference in %CD4^+^ cells (15+4.5% in all groups), or of Foxp3^+^ T cells (1.6+0.6% of CD4^+^ cells), in LP cells of mice from the 4 groups. In studies where WT, CD200^tg^ and CD200KO or CD200R1KO mice were injected with anti-CD4 mAb or isotype control Ig at days 4 and 7 post DSS initiation, anti-CD4 mab had no significant effect on weight loss in any groups (compare right and left hand panels in Fig 3E) with ≥96% depletion of CD4^+^ cells in all cases.

### CD200 reduced severity of DSS-induced chronic colitis

**Fig 4:**
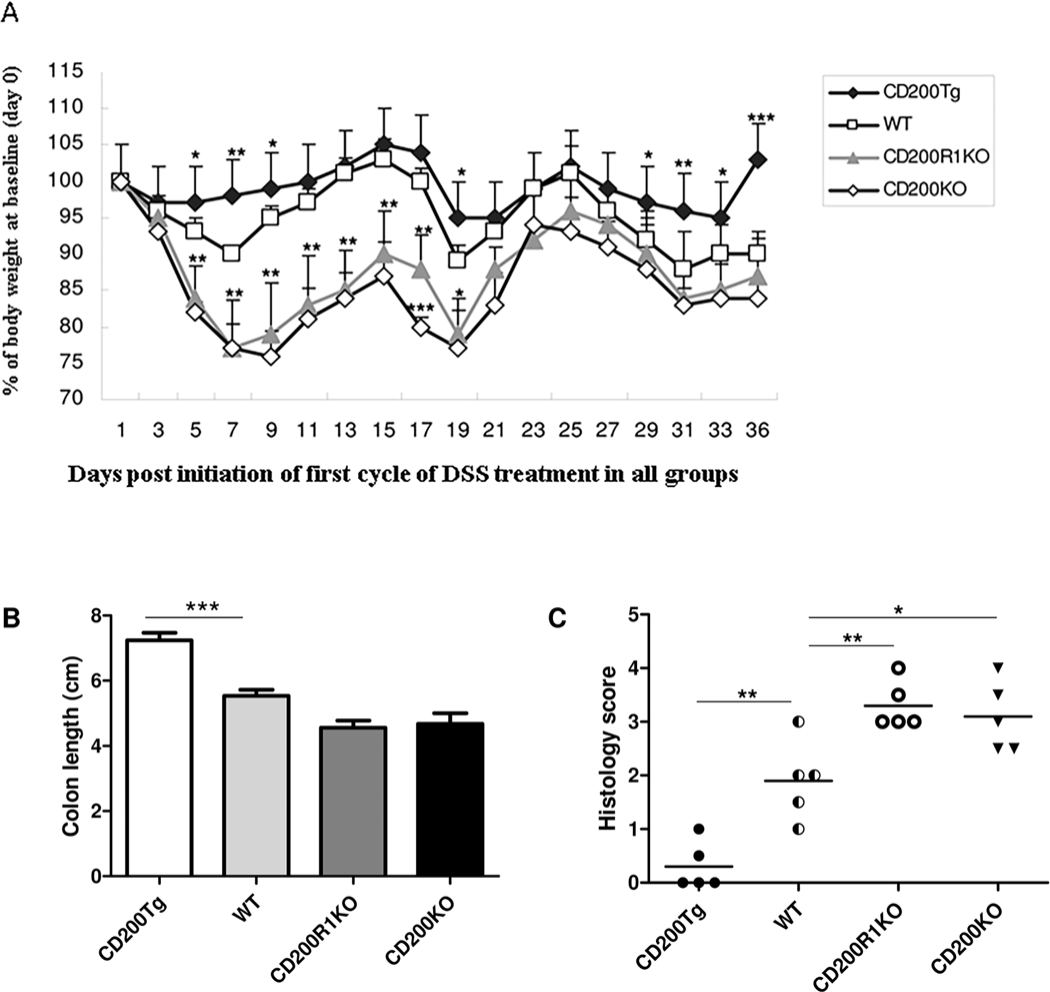
Changes in body weight, colon length and histological score of CD200^tg^, WT CD200R1KO and CD200KO mice with DSS-induced chronic colitis. (A) Change in body weight. Percent weight loss from baseline (y-axis) is plotted versus time post initiation of first cycle of DSS (x-axis). Data points represent mean ± SD for 5 mice. (B) Change in colon length assessed on 8 days post initiation of the third cycle of DSS for same mice. Data are expressed as mean ± SD of five mice. (C) Change in histological score (again 8 days post start of the third cycle of DSS) for same mice. All independent experiments were performed three times, yielding similar results. * p < 0.05; ** p < 0.01; *** p < 0.001 compared with WT controls on the same day-see also Fig 1.

Since human IBD is a chronic, relapsing inflammatory condition of the colon, we asked whether CD200 might also regulate DSS- induced chronic inflammation. The same four groups of mice were treated for 3 cycles (see Methods) with 5 days of DSS followed by 7 days of normal drinking water. Body weight changes in these mice are shown in Fig 4A. In all four groups a loss of weight occurred by days 7, 19 and 31, although the CD200^tg^ mice lost only 5% of body weight during that period. By day 36 these mice had gained ~ 5% over their initial body weight. In contrast, WT, CD200R1KO and CD200KO mice lost ~10–12% and ~13–25% of body weight respectively over this time. More significant differences were seen in the change of colon length among the four groups in this chronic colitis model compared with those seen in acute model (Fig 4B). The average colon length was reduced by ~12 mm in WT mice and by ~29 mm in CD200R1KO and CD200KO mice, compared with CD200^tg^ mice, with corresponding attenuation of histological scores (Fig 4C) in CD200^tg.^

### CD200 reduced histological changes and inhibited inflammatory response in chronic colitis

**Fig 5:**
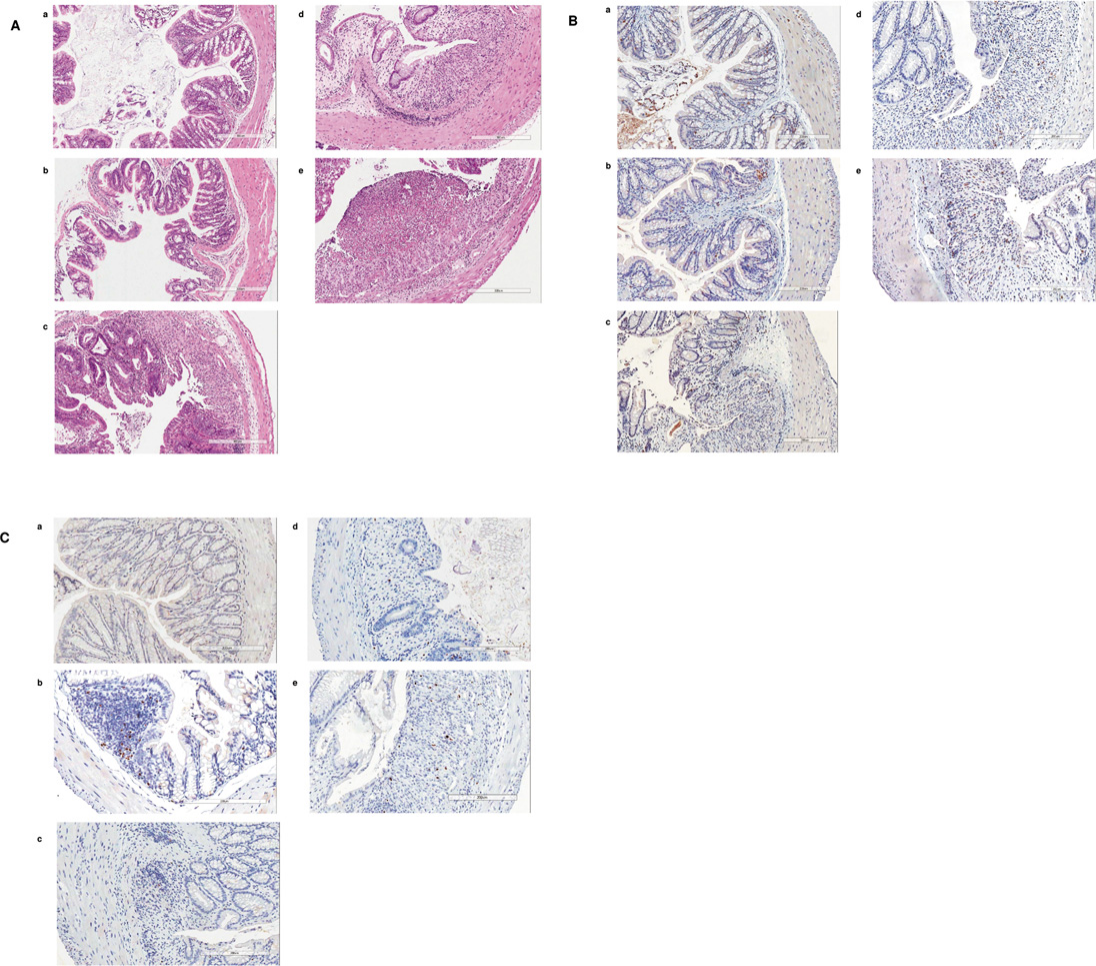
Effect of CD200 on histological changes and cell infiltration in colons from mice with DSS-induced chronic colitis. Sections from colon (8 days post start of the third cycle of DSS) of control (*a*), CD200^tg^ (*b*), WT (*c*), CD200R1KO (*d*) and CD200KO (*e*) mice were stained with (A) H&E, or with (B) rat anti-mouse CD3, or (C) rat anti-mouse Foxp3 mAbs, respectively-see also Fig 2. Percent staining in tissue is shown in Table 3 using counts of 1000 cells for each group (5 slides/group).

Figs 4C and 5A document that over-expression of CD200 protects from induction of histological changes associated with chronic colitis in CD200^tg^ mice compared with untreated control mice. Significant histological changes were still identified in the colons of WT, CD200KO and CD200RKO groups, with mononuclear leucocyte infiltration, crypt architectural disarray, transmural inflammation and severe edema (Fig 5A).

Immunohistochemistry staining using antibodies to cell surface markers revealed that myeloid cell infiltration persisted in WT, CD200R1KO and CD200KO mice, with few changes seen in CD200^tg^ mice (data not shown). However, unlike in acute colitis, chronic colitis has been reported to be associated with diffuse T cell infiltrates [31], and consistent with this view, tissues stained with anti-CD3 Ab showed more CD3^+^ cells infiltrating the colons of DSS-treated WT, CD200R1KO and CD200KO mice compared to CD200^tg^ mice (Fig 5B). Unlike observations in the acute colitis model described earlier, comparison of staining with anti-CD3 and anti-Foxp3 antibody (Fig 5C) revealed an increased Foxp3:CD3 ratio in the colon of CD200^tg^ mice compared with the other three groups (Table 2).

### Effect of CD200 on cytokine production in DSS-induced chronic colitis

**Fig 6:**
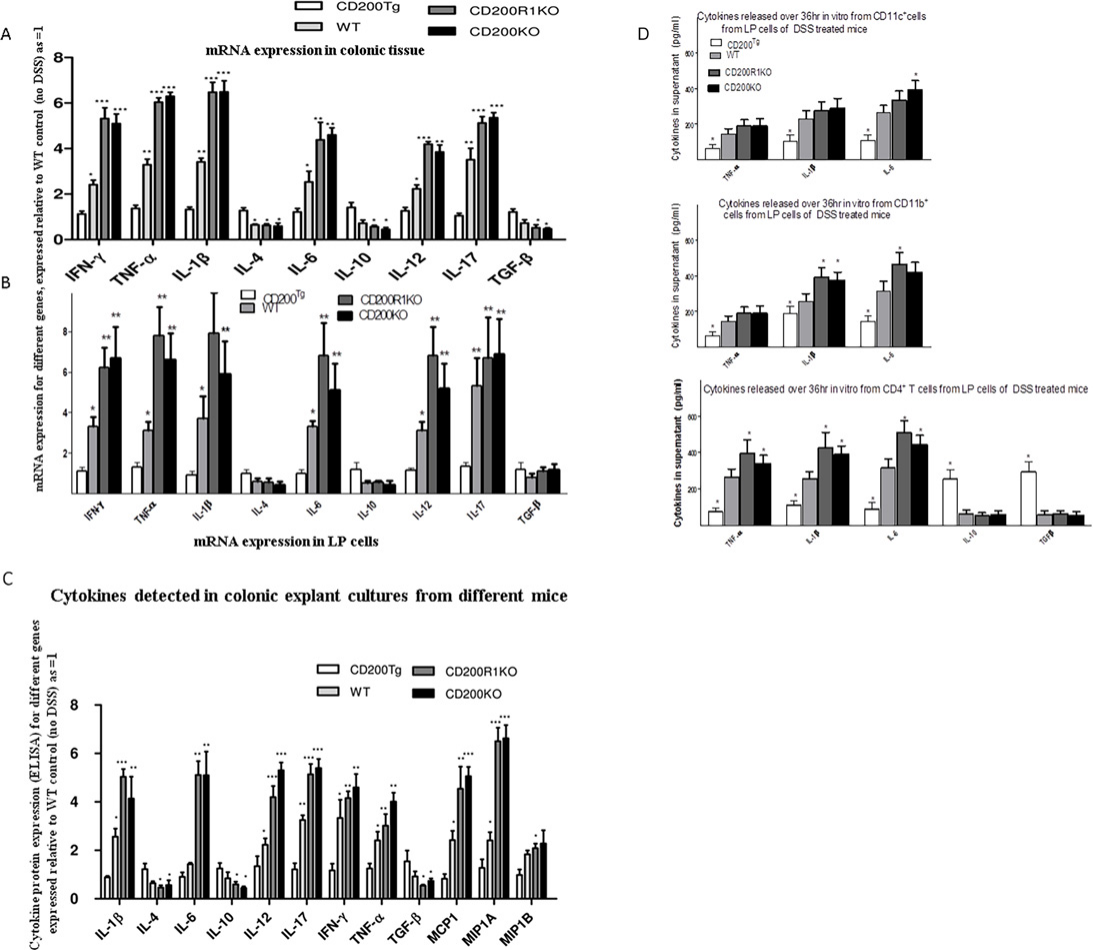
Effect of CD200 on expression of multiple cytokines/chemokines in DSS-induced chronic colitis. mRNAs for cytokines and chemokines were analyzed in colonic tissue harvested 8 days after initiation of the third cycle of DSS-induced chronic colitis from CD200^tg^, WT, CD200R1KO and CD200KO mice. Real-time RT-PCR was performed using total RNA extracted from colonic tissue (panel A: 5 samples/group) or LP cells (panel B: 3 samples/group), with data normalized to the expression of GAPDH and HPRT in the same organ. Expression levels in CD200^tg^, CD200R1KO and CD200KO mice (mean ± SD in 5 mice) are shown relative to that in WT at day 0 (designated as 1). * p < 0.05; ** p < 0.01; *** p < 0.001, compared with WT control (day 0). (C) Tissue explants from the four experimental groups were cultured and used for multi-analyte Elisarray. Again, relative expression levels of cytokine/chemokine (mean ± SD in 5 mice) in CD200^tg^, CD200R1KO and CD200KO mice are shown compared to that in WT at day 0 (designated as 1), using manufacturer’s software. * p < 0.05; ** p < 0.01; *** p < 0.001 compared to WT control (day 0). (D) Inflammatory cytokines measured in 36hr culture supernatants of colon-derived CD11c^+^, CD11b^+^, or CD4^+^ T cells (isolated by MACS columns) from WT, CD200^tg^, CD200KO or CD200RKO mice 8 days post initiation of the third cycle of DSS treatment. Cells were obtained from a pool of 4 mice/group, and cultured without further stimulation for 36hrs in complete medium. Data show mean (+SD) of triplicate measurements. *, indicates p<0.05 compared with WT control-see also Fig 3D.

Colonic cytokine expression patterns were compared among the four experimental groups to assess the influence of CD200 over-expression on cytokine production in chronic colitis. Compared to WT mice, whether analyzed by mRNA (Fig 6A and 6B) or multi-analyte protein expression (Fig 6C), both CD200R1KO and CD200KO groups showed higher levels of expression of IFN-γ, IL-17, IL-1β and MIP1A and lower levels of IL-4, IL-10 and TGF-β, while the CD200^tg^ group showed significantly elevated levels of IL-4, IL-10 and TGF-β with lower levels of IFN-γ, IL-17, TNF-α, IL-1β, MIP1A and 1B. These data are again consistent with the hypothesis that amelioration of DSS-induced chronic colitis by CD200 occurs in association with of polarization of cytokine production to type-2 cytokines. Cytokine production from MACS- column enriched CD4^+^ cells isolated from LP cells in this chronic colitis model (Fig 6D) contrasted with data from mice with acute colitis (see Fig 3D). In WT and CD200KO and CD200R1KO mice, CD4^+^ cells were now a prominent source of IL-1b, TNFa and IL-6, in contrast to cytokine production from CD4^+^ cells in mice with acute colitis (Fig 3D). Indeed in chronic colitis, relatively equivalent cytokine levels were observed in CD4^+^ and CD11c^+^, CD11b^+^ cells (Fig 6D).

**Fig 7:**
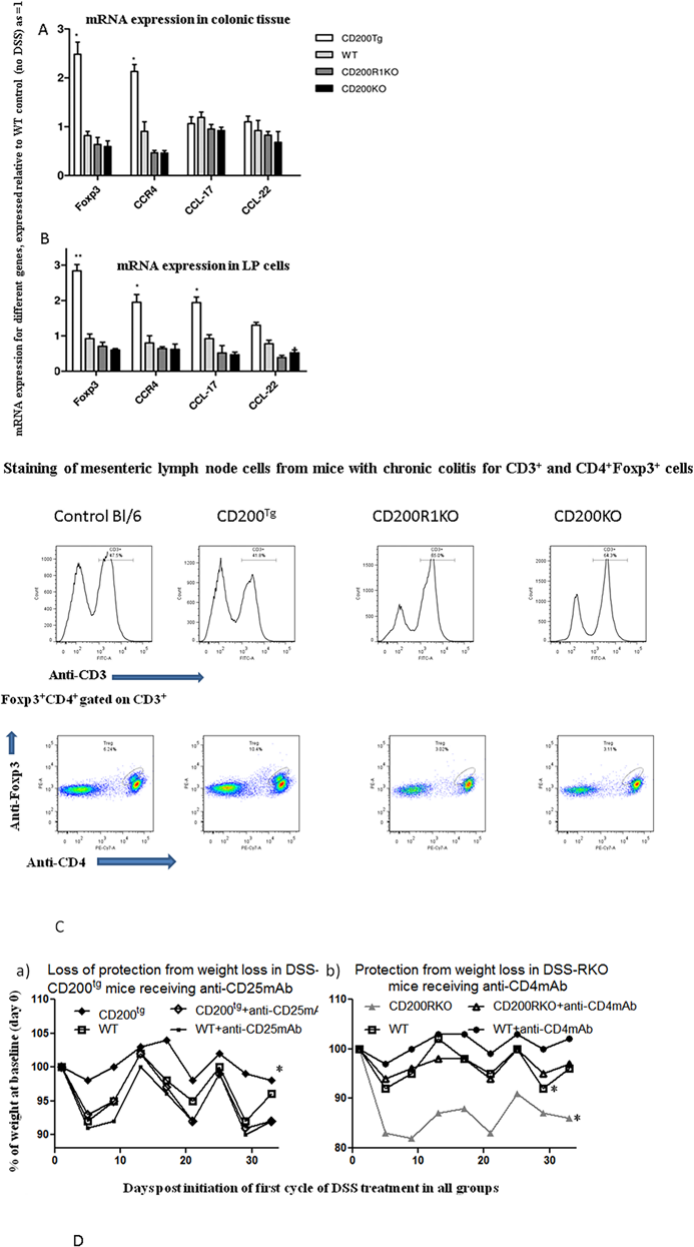
The effect of CD200 on regulation of expression of CCR4 and Foxp3 in DSS-induced chronic colitis, and importance of CD25^+^Tregs and CD4^+^ T effector cells to DSS-induced weight loss. Foxp3, CCR4, CCL-17 and CCL-22 mRNA expression was analyzed in (A) colons and (B) LP cells by real-time RT-PCR from CD200^tg^, WT, CD200R1KO and CD200KO mice with DSS-induced chronic colitis. All values were normalized to the expression of GAPDH and HPRT in the same organ with expression levels in CD200^tg^, CD200R1KO and CD200KO mice expressed relative to that of WT (designated as 1). Data show mean ± SD for 5 mice/group. * p < 0.05; ** p < 0.01. (C) FACS staining for CD3^+^ and CD4^+^Foxp3^+^ (gated on CD3^+^ cells) in mesenteric lymph node (MLN) cells of mice in panels A and B. Data show typical staining patterns from pooled samples (3 mice/group). (D) Loss of protection from weight loss in DSS-induced chronic colitis in CD200^tg^ mice by treatment with anti-CD25mAb (Fig 7Da), and protection from weight loss in CD200RKO mice by treatment with anti-CD4 mAb (Fig 7Db). Data are shown for 4 mice/group (SD not shown to retain clarity in panels). Mice received 5mg antibody iv 1 day following completion of each DSS treatment. *p<0.05 (Mann-Whitney U-test) compared with equivalent groups not receiving mAb.

Given earlier studies showing that CD200 regulated expression of the chemokine receptor CCR4, an attractant to Treg cells [32], we examined expression levels of CCR4 and two CCR4 ligands, CCL-17 and CCL-22 in CD200^tg^ mice. We observed increased expression in both the colon and LP cells (Fig 7A and 7B). FACS analysis of mesenteric lymph node cells isolated from the different groups revealed increased numbers of Foxp3^+^CD4^+^ cells in CD200^tg^ mice (Fig 7C), and an increased Foxp3:CD3 ratio, consistent with immunohistochemistry of isolated colonic tissue (scoring slides for both Foxp3^+^ and CD3^+^ staining) which had revealed an increased Foxp3:CD3 ratio in the CD200^tg^ group compared with all other groups (see Table 3).

**Table 3:** Quantitation of staining of CD3^+^ and Foxp3^+^ cells in slides shown in Fig 5B and 5C.

Cell staining	Control	CD200^tg^	WT	CD200R1KO	CD200KO
%anti-CD3	3±2	8.0±2	15±4*	33±6**	28±5**
%anti-Foxp3	0.1±0.1	0.5±0.2	0.3±0.2	0.5±0.3	0.5±0.2
Foxp3:CD3	0.03*	0.06	0.02*	0.02*	0.02*

*P<0.05 compared with CD200^tg^

** p<0.05 compared with WT

***Footnote*:** Colonic sections from 5 different groups of mice were stained with anti-CD3 or anti-Foxp3 antibodies. Percent cells stained was enumerated by counting (5 slides/group) a total of 1000cells.

Finally, in Fig 7D, we investigated the effect of infusion of mAbs designed to deplete CD4^+^ T cells (GK1.5) or CD25^+^ Tregs (PC61) on weight loss in WT/CD200R1KO or CD200^tg^ mice respectively, treated using this chronic DSS protocol (see also Fig 3E). In this instance, mice received.3 infusions of 50mg Ig iv one day following completion of each DSS cycle. It is apparent from these studies that depletion of CD25^+^Tregs (confirmed by >80% decrease in Foxp3^+^ cells staining in colonic tissue compared with isotype Ig controls) in CD200^tg^ mice led to attenuation of protection from weight loss afforded by CD200 over-expression (Fig 7Ga). In contrast, depletion of CD4^+^ cells from both WT and CD200R1KO mice, documented in Fig 6D to represent a major source of inflammatory cytokines in chronic colitis, improved weight gain in these animals, most markedly for CDR00RKO mice (see Fig 7Gb). Taken together, these data support the idea that over-expression of CD200 in CD200^tg^ mice enhances CCR4 dependent Foxp3^+^ Treg cell migration to the colon in mice to suppress a chronic inflammatory response associated with chronic DSS colitis. Further, that in the absence of regulation by CD200:CD200R interactions, CD4^+^ T cells over-producing inflammatory cytokines contribute significantly to pathology.

## Discussion

Loss of an intact mucosal barrier and dysregulated immune responses to common luminal bacterial antigens are thought to precede the development of IBD. DSS is directly toxic to the colonic epithelial cells of the basal crypts and thus can break down the function of the mucosal epithelial barrier, resulting in the entry of luminal microorganisms into the mucosa where an inflammatory response is triggered [18,33]. Consistent with the notion that regulation of the inflammatory response is key to the pathogenesis of IBD are data reporting a role for Toll-like receptors (TLRs) in disease control [34,35]; of Nlrp3, and the “inflammasome” in DSS-induced colitis [36]-see also [37]; and of dysregulation of inflammatory cytokines in disease [38–42]. While the DSS-induced colitis model has been a popular one for investigators exploring factors implicated in human disease [43], there are significant variables which may contribute to a complexity in generalizing data from murine DSS studies to humans, and even in comparing different murine DSS studies with one another. Responses to DSS are dependent not only on the DSS used and its application (i.e. concentration, molecular weight, duration of DSS exposure, manufacturer, and batch) but also on the host genetic background; the intestinal microbiota in the animals used for initiation of disease [33]; and background stress levels in the host animal (colony) [44]. While mucosal destruction seen after DSS treatment occurred even in the absence of contributions from the intestinal microbiota, the latter were clearly implicated in the susceptibility and responsiveness of epithelial cells to DSS-induced pathology [33]. In addition, mice deficient for different toll-like receptors (TLR), showed markedly different susceptibilities to DSS-colitis [33,45–48].

Our studies investigated whether over-expression of CD200 could reduce inflammatory reactions in the colon and attenuate the severity of DSS-induced colitis under acute and/or chronic conditions in mice. We show that over-expression of CD200 protected mice from DSS-induced acute or chronic colitis, and in our hands mice lacking a functional CD200 signal (CD200KO or CD200R1KO mice) displayed more severe clinical and histological changes compared with WT mice. Interestingly, a previous study using mice derived from these same founder stock came to the conclusion that CD200R1KO mice were *not* more susceptible to DSS-induced acute colitis than WT mice [49]. However, as noted above (see also [33]), the response to DSS is recognized to be dependent on many factors. Without direct comparison of the source of the DSS itself; any potential genetic drift in the mice used; and the host microbial microbiota, it remains unclear as to why the Bain et al studies reported different findings. However, given the symmetry we observe in the decreased susceptibility to colitis in CD200^tg^ mice, with increased susceptibility in CD200KO and CD200RKO mice, along with evidence that in vitro inflammatory cytokine production from colonic DCs and macrophages of both WT and CD200KO mice is attenuated by exogenous CD200Fc (Fig 3D), our data argue strongly that the CD200:CD200R axis plays a prominent role in regulating inflammatory DSS-induced acute colitis in mice. In regard to the contribution of host microbiota on DSS-induced colitis, it is important to note that all mice used in our study were treated with a chronic regimen of doxycycline (needed to induce CD200-transgene expression in CD200^tg^). However, we have observed no significant different (gross colonic pathology; immunohistology; cytokine production) in colonic disease in WT, CD200KO or CD200R1KO animals maintained on either doxycycline or plain drinking water, implying that an impact of doxycycline on the intestinal microbiotia is unlikely to represent an important variable leading to differences between our own and previously published data, or to differences in disease between the various strains of mice. The failure of exogenous CD200Fc to reduce in vitro inflammatory cytokine production by colonic CD11c^+^ or CD11b^+^ cells of DSS-treated CD200RKO mice (Fig 3D) is consistent with our report elsewhere concerning the immunobiology of these CD200R1KO mice and their resistance to reduction of inflammation in the presence of elevated levels of CD200 [23].

The most strongly expressed inflammatory cytokines in acute colitis seen in CD200R1KO and CD200KO (but not CD200^tg^) mice were produced by cells of the myeloid lineage (CD11c^+^ and CD11b^+^) and not T cells (Fig 3D). These data are consistent with previous evidence from Hoek, et al. that CD200 exerts an inhibitory function on myeloid cells [14], and that absence of CD200 signaling contributes to polarization of cytokine production towards an inflammatory cytokine profile [28]. Acute colitis is thought to be Th1/Th17 mediated [38], and our data in the acute colitis model described above shows a heightened Th1/Th17 response in both CD200R1KO and CD200KO mice, with decreased expression of mRNA and protein expression for Th1 cytokines and increased expression of Th2 cytokines in CD200^tg^ mice (Fig 3). This may help explain why more severe colitis was seen in CD200R1KO and CD200KO mice compared with the WT group. Importantly, cells of the myeloid lineage, and not T cells, were the primary source of inflammatory cytokines in this acute colitis model (Fig 3D), and depletion of CD4^+^ cells by antibody (starting at day 4 post DSS treatment) failed to alter acute DSS disease outcome in the various groups (Fig 3E), consistent with the hypothesis that Foxp3^+^CD4^+^ Tregs were not associated with disease outcome.

In chronic colitis, by contrast, in addition to myeloid cell infiltration, an increased CD3^+^ cell infiltration was observed in the colon (Fig 5B) which was ameliorated in CD200^tg^ mice by over-expression of CD200 (Figs 4 and 5). The cytokine production profile observed in tissues with chronic colitis again showed increased expression of IFN-γ and IL-17 in CD200R1KO and CD200KO mice compared with either CD200^tg^ or WT mice (Fig 6A–6C), consistent with reports on the role of IFN-γ and IL-17 in DSS-induced chronic colitis in mice [31,50]. However, unlike in the acute colitis model, CD4^+^ T cells in this chronic colitis model were an important source of inflammatory cytokines (compare Figs 3D and 6D).

There has been interest in the role of other cells besides DCs [30], macrophages and T cells in colitis in humans and animal models. Mizoguchi et al [51], using TCRα^-/-^mice, reported data implicating a role for mature B cells in the development of chronic colitis by directly regulating functional activity in pathogenic T cells (CD4^+^ TCRα^-^β^+^ T cells). B cells are known to be a potential source of inhibitory cytokines such as IL-10 and TGF-β, while others have also reported that under different signaling conditions, pro- or anti-inflammatory cytokines can be produced [52]. We have not assessed a role for B cells in either the acute or chronic colitis models discussed above. Group 3 ILCs (ILC3), secreting IL-17 and IL-22 and expressing the transcription factors RORγt and AHR regulate intestinal immunity [6,7,29], and preliminary analysis of altered mRNA expression characteristic of such cells (S1 and S2 Figs) in tissue from acute/chronic colitis mice are consistent with their also playing a role in this model.

Defects in Foxp3^+^Treg cells, known to play a role both in the maintenance of tolerance and suppression of inflammation, have been implicated in the pathogenesis of IBD [53–56]. In a mouse model of IBD Tregs served a role in attenuating disease, a suppressive function which was abolished in the absence of CCR4, a chemoattractant for such cells [32,57]. Eijkelkamp et al [58] reported an increase in Foxp3 (detected by Western blot) in colons of WT mice in an acute colitis model which resolved by ~day 70 post induction with DSS. A GRK6^-/-^ mouse, with a propensity to develop chronic colitis, showed attenuated Foxp3 at d16, which the authors speculated may have attributed to the chronic disease development-there was also evidence in this study, however, that Foxp3^+^Tregs in the colonic tissue of acute/chronic colitis mice were not functionally equivalent. In our studies we observed no differences in Foxp3/CCR4 mRNA expression in colonic tissue amongst the four groups of mice in an acute colitis model, despite differences in disease by histopathology (Fig 2) and observed no attenuation of protection from DSS-induced acute colitis in CD200^tg^ mice following depletion of CD4^+^ cells (Fig 3E). However, in the chronic colitis model, increased expression of both CCR4 and Foxp3 was detected in CD200^tg^ mice (Fig 7A and 7B), with an increased ratio of Foxp3^+^:CD3^+^ cells enumerated in the same CD200^tg^ mice both in colonic tissue sections (Fig 5 and Table 2) and by FACS staining of isolated mesenteric lymph node cells (Fig 7C). We hypothesize that in CD200^tg^ mice over-expression of CD200 contributes to attraction of Tregs through up regulation of CCR4, and these Tregs help to restore homeostasis. Importantly, infusion of anti-CD25 to deplete Tregs abolished protection by CD200 over-expression in CD200^tg^ mice with DSS-induced chronic colitis (Fig 7Da-compare with Fig 3E). However, the functional activity of the Tregs (in terms of their suppressive activity) was not explored in this chronic DSS model, nor have we compared Treg function in mice with acute/chronic DSS-colitis. The importance of Teffector cells in chronic colitis was also implied from data showing that infusion of anti-CD4 mAb into CD200KO and CD200RKO mice improved weight gain in these mice (Fig 7Db). The interpretation of the effects seen with anti-CD25 and anti-CD4 mAbs is marred by potential cross depletion of Teff or Tregs by each mAb, although the difference between the effects of anti-CD4 (improves health) and anti-CD25 (worsens disease) in Fig 7 lessens this concern regarding such cross-reactivity.

In summary, our data argue strongly for an important role for CD200 overexpression in regulation of gut inflammation, in animals suffering from both acute and chronic colitis. In an acute model, CD200 can attenuate inflammatory cytokine production by myeloid cells, which likely represents a key contributor to the pathophysiology of disease. In contrast, in mice with chronic colitis, CD200 overexpression seems to exert a more important role in regulating Foxp3^+^ Treg levels/function to regulate colitis, likely through altering the molecular milieu (cytokines/chemokines) to regulate numbers/localization of Tregs. Current treatments for human IBD mainly rely on immunosuppressive drugs and are not curative. Our findings provide insight into a potential novel alternative strategy for treatment of human IBD.

## Supporting Information

S1 Fig Effect of CD200 on expression of ILC related transcription factors in DSS-induced acute colitis. Real-time RT-PCR was performed using total RNA extracted from colonic tissue (5 samples/group) or LP cells (3 samples/group), with data normalized to the expression of GAPDH and HPRT in the same organ. Relative expression levels of ILC related transcription factors in CD200^tg^, CD200R1KO and CD200KO mice are shown compared to that in WT at day 0 (designated as 1). * p < 0.05; ** p < 0.01; *** p < 0.001, compared with WT control (day 0). See also legend to Fig 3.
(TIF)

S2 Fig Effect of CD200 on expression of ILC related transcription factors in DSS-induced chronic colitis. mRNAs for transcription factors were analyzed in colonic tissue harvested 8 days after initiation of the third cycle of DSS-induced chronic colitis. from CD200^tg^, WT, CD200R1KO and CD200KO mice. Real-time RT-PCR was performed using total RNA extracted from colonic tissue or LP cells, panels (A) and (B) respectively, with data normalized to the expression of GAPDH and HPRT in the same organ. Relative expression levels of ILC related transcription factors in CD200^tg^, CD200R1KO and CD200KO mice are shown compared to that in WT at day 0 (designated as 1). * p < 0.05; ** p < 0.01; *** p < 0.001, compared to WT control (day 0). See also legend to Fig 6.
(TIF)

## Abbreviations

DSS: Dextran Sodium Sulfate
IBD: inflammatory bowel disease
Dox: doxycycline
CD200^tr^: CD200 transgenic
LP: Lamina Propria
IL: interleukin
Th: T helper
Treg: regulatory T cell
ILC: innate lymphoid cell
RORγt: retinoic acid-related orphan receptor γt
