# Supplementary figures and images for: Over-Expression of CD200 Protects Mice from Dextran Sodium Sulfate Induced Colitis
Source: PLoS One. 2016 Feb 3;11(2):e0146681. doi: 10.1371/journal.pone.0146681 (PMC4740450; doi:10.1371/journal.pone.0146681)

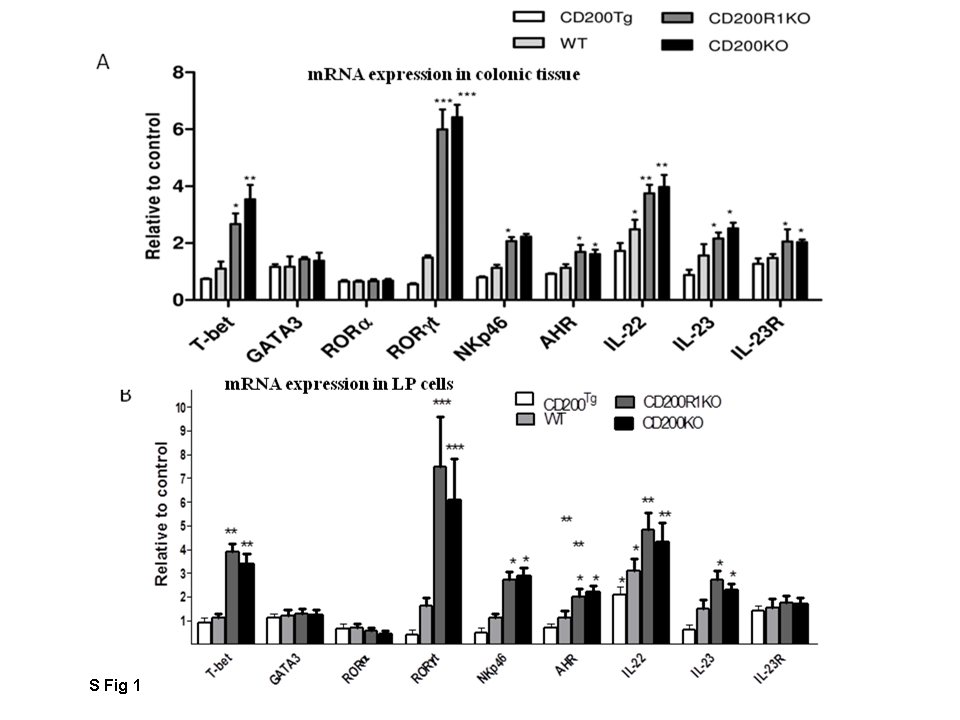

Supplement: S1 Fig — Real-time RT-PCR was performed using total RNA extracted from colonic tissue (5 samples/group) or LP cells (3 samples/group), with data normalized to the expression of GAPDH and HPRT in the same organ. Relative expression levels of ILC related transcription factors in CD200tg, CD200R1KO and CD200KO mice are shown compared to that in WT at day 0 (designated as 1). * p < 0.05; ** p < 0.01; *** p < 0.001, compared with WT control (day 0). See also legend to Fig 3. (TIF) [file pone.0146681.s001.tif]

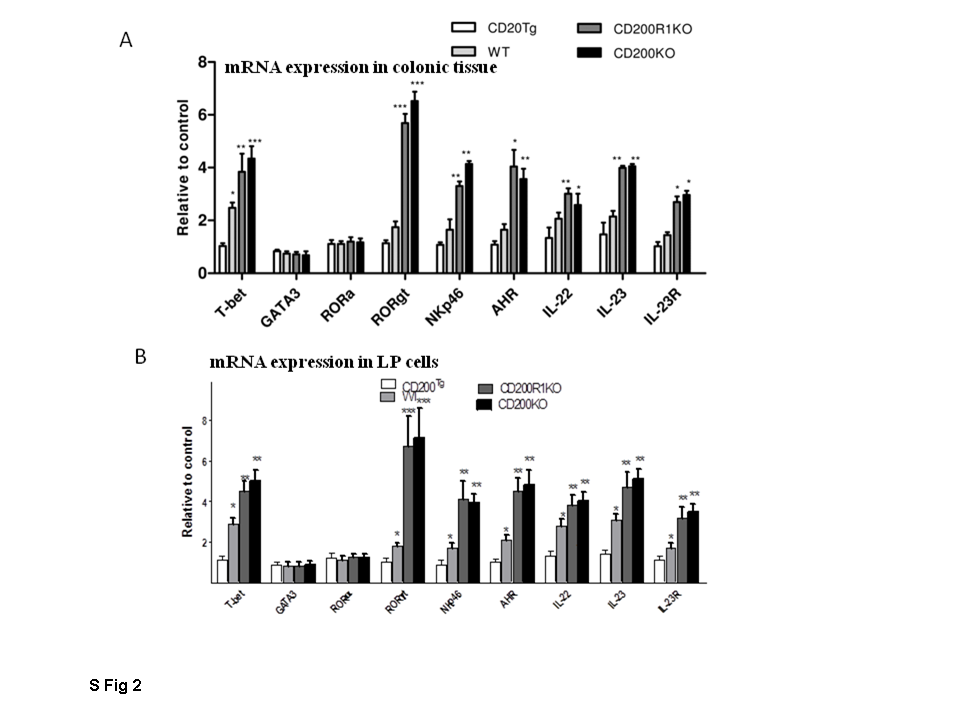

Supplement: S2 Fig — mRNAs for transcription factors were analyzed in colonic tissue harvested 8 days after initiation of the third cycle of DSS-induced chronic colitis. from CD200tg, WT, CD200R1KO and CD200KO mice. Real-time RT-PCR was performed using total RNA extracted from colonic tissue or LP cells, panels (A) and (B) respectively, with data normalized to the expression of GAPDH and HPRT in the same organ. Relative expression levels of ILC related transcription factors in CD200tg, CD200R1KO and CD200KO mice are shown compared to that in WT at day 0 (designated as 1). * p < 0.05; ** p < 0.01; *** p < 0.001, compared to WT control (day 0). See also legend to Fig 6. (TIF) [file pone.0146681.s002.tif]
